# Investigation of answer changes on the USMLE® Step 2 Clinical Knowledge examination

**DOI:** 10.1186/s12909-019-1816-3

**Published:** 2019-10-23

**Authors:** Wenli Ouyang, Polina Harik, Brian E. Clauser, Miguel A. Paniagua

**Affiliations:** National Board of Medical Examiners, 3750 Market Street, Philadelphia, PA 19104 USA

**Keywords:** Step 2 CK, Answer change on MCQ, USMLE, Test taking, Credentialing examination

## Abstract

**Background:**

Examinees often believe that changing answers will lower their scores; however, empirical studies suggest that allowing examinees to change responses may improve their performance in classroom assessments. To date, no studies have been able to examine answer changes during large scale professional credentialing or licensing examinations.

**Methods:**

In this study, we expand the research on answer changes by analyzing responses from 27,830 examinees who completed the Step 2 Clinical Knowledge (CK) examination between August of 2015 and August of 2016.

**Results:**

The results showed that although 68% of examinees changed at least one item, the overall average number of changes was small. Among the examinees who changed answers, approximately 45% increased their scores and approximately 28% decreased their scores. On average, examinees spent shortest time on the item changes from wrong to right and they were more likely to change their scores from wrong to right than right to wrong.

**Conclusions:**

Consistent with previous studies, these findings support the beneficial effects of answer changes in high-stakes medical examinations and suggest that examinees who are overly cautious about changing answers may put themselves at a disadvantage.

## Background

There are, no doubt, many areas where conventional wisdom stands in sharp contrast to a substantial body of empirical evidence. In the field of assessment, there is probably none more notable than the “conventional wisdom” that examinees should go with their first impressions and avoid changing answers on multiple choice tests. Numerous authors spanning more than 75 years have reported on examinee beliefs that changing answers is likely to negatively impact their scores. One early study [19] reported that 86% of students believed that changing answers would not benefit their scores; more recently Geiger [12] and Kruger, Wirtz and Miller [18] reported that a substantial percentage of examinees continued to believed that changing answers would lower their scores. Professionally developed test preparation materials additionally seem to support the view that considerable caution should be exercised in changing answers, for example, *How to Prepare for the GRE: Graduate Record Examination* [5] and *First Aid for the USMLE Step 1* [3] both include a recommendations of this sort.

The impressions of students and recommendations from test preparation guides aside, there have been numerous empirical studies examining the impact of changing answers and these studies consistently support the benefit of changing answers (e.g., [1, 2, 6, 8–10, 13–15, 17, 20, 21, 24–26, 29]; for a meta-analysis and review, see [27]). The general conclusion from these studies is that given time for review, most examinees change answers, but on average examinees make changes to a small number of items. Additionally, changes typically are from wrong to right answers more often than from right to wrong. Examinees increased their scores 53% of the time by changing their answers according to one study [19] and 51% of the time according to another [18]. These results seem to be typical of the available literature on answer changes. Moreover, previous studies have suggested that both higher and lower performing students benefit from answer-changing, and higher performing students might gain more points through answer-changing than lower performing students [7, 11, 16, 22, 23]. To our knowledge, there is only one published study which argues that answer changes could be detrimental [28]. However, these authors have subsequently published a retraction of the initially reported results owing to an error with the data analysis. Bridgeman [4] subsequently reanalyzed the data and reported that 76% of examinees improved their scores by changing answers.

The results of previous studies seem to be consistent, but many of these studies are based on the behavior of college psychology or nursing students completing classroom course assessments. These results may—or may not—generalize to professionally developed, high-stakes examinations such as selection (e.g., the SAT and GRE) or credentialing examinations (e.g., USMLE). For these latter types of examinations, examinees frequently engage in extensive test preparation which may influence examinee behavior in ways that could lead to different outcomes. Additionally, the test development procedures used at major testing organizations for developing high-stakes tests may be substantially different than those used in developing classroom assessments; these differences could impact the utility of answer changes. Two recent studies have provided some information about answer changes on these types of examinations. A small scale study by Fischer et al. [9] examined answer changes of 36 students on the German Second National Medical Board Examination. Results showed that test scores improved when students changed their answers once (although further answer changes did not improve the score). A more recent study [24] of answer changes for the Graduate Record Examination (GRE) similarly confirmed the potentially beneficial effects of changing answers. This study further examined how answer change benefits varied as a function of examinee ability reporting that the benefits of answer change increase as the examinee’s ability increases.

The United States Medical Licensure Examination (USMLE®) is a three-step medical examination system required for all physicians with an MD degree seeking licensure in the United States. The USMLE assesses how well medical graduates can apply their attitudes, skills, and values to real patient-centered scenarios. Thousands of U.S. and international medical students take the USMLE every year; the resulting data provide a unique opportunity to examine the potential benefits of answer change in a very large sample of high-stakes examination. However, no published studies have examined answer changes on USMLE. The present study fills this gap in the literature with a large scale study of the effects of answer changes on the USMLE Step 2 Clinical Knowledge (CK) examination, the second part of USMLE sequence. The present study follows the example of the Liu et al. [24] study by considering examinee ability as a factor in analyzing answer change results. This study also extends the literature related to answer changing by considering how patterns of answer changes vary as a function of both item difficulty and the amount of time examinees spent on responding to an item. In addition to these general results which expand our understanding of examinee test taking strategies and behavior, the present paper provides results that may be practically useful for students preparing for USMLE and other medical credentialing examinations and educators who support that preparation.

## Methods

### Examinee sample

We analyzed responses from 27,830 examinees comprising an annual cohort that completed the Step 2 CK examination between August of 2015 and August of 2016. Again, the Step 2 CK examination is part of the sequence of examinations required for physician licensure in the United States. Nearly 90% of examinees in the sample took the Step 2 CK for the first time, 86.7% passed the exam, 66% were native speakers of English and 54.4% were male. Table 1 provides more complete descriptive information about the examinee sample.

**Table 1:** Examinee sample description (*N* = 27,830)

	Number of examinees	%	Step2 CK score mean (SD)
Gender			
Male	15,151	54.4%	232 (22.8)
Female	12,679	45.6%	233 (22.6)
English proficiency			
ESL	9474	34.0%	226 (23.9)
ENL	18,356	66.0%	237 (21.0)
Number of takes			
First time takers	24,939	89.6%	236 (21.5)
Repeaters	2891	10.4%	210 (18.6)
Pass/Fail			
Pass	24,119	86.7%	239 (16.1)
Fail	3711	13.3%	192 (16.4)
Total	27,830		233 (22.7)

### Data

At the time the data were collected the Step 2 CK examination consisted of eight one-hour blocks with 40–45 multiple choice items administered in each block. The test is computer administered and examinees are able to return to items within a block, but they cannot return after they have completed a given block. The data collected for this study included detailed information about each action an examinee took while navigating through a block of items, such as time spent on each item, the sequence in which the examinee visited and revisited each item, and the response to each item on each visit. Because the dataset containing information about each action was large and complex, we focused on two of the eight hour-long blocks for this study. We selected blocks four and eight. We believed that block four would be typical of examinee behavior throughout the test day; we included block eight to confirm that behavior did not change substantially across the day. Although previous unpublished analysis has shown that any effects of fatigue across the test day are negligible, we believed that if there were changes across the test day they would likely be most apparent in the last block. The data set additionally included examinee biographical information, overall Step 2 CK scores (μ = 233, sd = 22.7) and item difficulties (μ = 0, sd = 1.4) based on one-parameter Item Response Theory (IRT) calibration.

### Analyses

For each examinee, we computed 1) the number of items revisited, 2) the number of revisited items with no response change, and 3) the number of revisited items with response changed from wrong to right (W-R), right to wrong (R-W), wrong to wrong (W-W) and right to right (R-R). Changes in responses were only considered for non-blank responses. Response changes were based on the examinees’ initial selected response and their final selected response. If an examinee changed their response multiple times, the intermediate responses were ignored except in the case of changes in which an examinee changed a correct answer to an incorrect answer during an intervening visit and then changed it back to a correct answer during the final visit. Such changes were considered right to right (R-R). Responses were only included when an examinee completed an item and moved to a different item, so response changes made within a single visit to an item were not considered. We used this rule to eliminate the impact of answer changes that were simply immediate corrections of typographical errors.

We additionally computed the percentage of examinees with score gains and score losses that resulted from answer changes and the W-R/R-W ratio. In addition to examining these results for the full sample, we examined how results varied as a function of examinee ability (low, medium and high based on their total Step 2 CK score). The low ability group comprised examinees who failed the examination. The medium ability group included examinees with passing scores within one standard deviation from the cut score. The high ability group included all examinees with scores more than one standard deviation above the cut score.

Multinomial logistic regression analysis was used to compare the odds of different change patterns for different examinee ability groups. The type of change (W-R, R-W, R-R, and W-W) was used as a dependent variable, ability group as independent variable and response duration as a covariate. R-W was specified as a reference category.

Finally, we examined how the time spent in reviewing items varied across 4 response change patterns (W-R, R-W, R-R, and W-W) using a linear mixed effects model. The type of answer change and English proficiency were treated as fixed effect factors. Difference among subjects was included as a random term. The model was adjusted for examinee ability and item difficulty.

Step 2 CK examinees have the opportunity to decline to have their data used for research. Less than .01% of examinees in this cohort declined. This study was reviewed by the American Institutes for Research Institution Review Board and qualified for exempt status because it involved very minimal or no risk to study subjects. All analyses were performed using PASW SPSS Statistics 23.0 (SPSS Inc., Chicago, IL, USA).

## Results

### Block four analyses

Most examinees (99.0%) revisited at least one item, and 68% of examinees changed at least one response. The overall response change patterns and outcomes are shown in Table 2. Among the examinees who changed responses 44.7% increased their scores and 27.8% lowered their scores.

**Table 2:** Response change patterns and outcomes for block 4

	Number of examinees	% (Based on all examinees)	% (Based on examinees with response change)
Examinees who revisited at least one item	27,560	99.0%	
Examinees who changed at least one response	18,936	68.0%	100%
*Examinees with score gain*	8461	30.4%	44.7%
*Examinees with score loss*	5263	18.9%	27.8%
*Examinees with unchanged score*	5212	18.7%	27.5%
	Mean number of items	% (Based on all items)	% (Based on items with revisits)
Items with revisits	16.0	36.4%	100%
Items with revisits and no response change	14.6	33.2%	91.2%
Items with revisits and response change	1.4	3.2%	8.8%
*W-R*	*0.59*		
*R-W*	*0.40*		
*R-R*	*0.03*		
*W-W*	*0.38*		
W-R/R-W ratio	0.59/0.40 = 1.48		
Overall mean score change	0.004		

*W-R* wrong to right response changes, *R-W* right to wrong response changes, *W-W* wrong to wrong response changes, *R-R* right to right response changes

On average each examinee re-visited 16 items. However, among these re-visited items, the majority (14.6) had no response change and on average each examinee only changed responses 1.4 items. Out of the 1.4 items with response change, 0.59 responses were changed from wrong to right, 0.40 were changed from right to wrong, 0.38 were changed from wrong to wrong, and 0.03 were changed from right to right. The W-R/R-W ratio was 1.48 (0.59/0.40).

Table 3 displays results analogous to those in Table 2, but separated by whether the examinees increased or decreased their scores as a result of answer changes. These results show only modest differences between the two groups, but suggest that examinees who improved their scores changed a slightly greater number of answers (2.2 ± 1.5 items as opposed to 2.0 ± 1.2).

**Table 3:** Response change patterns and outcomes by score change for block 4

	Score Gain (*n* = 8461)		Score Loss (*n* = 5263)		No Score Change (*n* = 5212)	
	Mean number of items	%	Mean number of items	%	Mean number of items	%
Items with revisits	18.6	42.3%	17.8	40.4%	13.8	31.3%
Items with revisits and no response change	16.4	88.2%^a^	15.8	88.8%^a^	13.1	94.8%^a^
Items with revisits and response change	2.2	11.8%^a^	2.0	11.2%^a^	0.7	5.1%^a^
*W-R*	1.55		0.14			
*R-W*	0.16		1.36			
*R-R*	0.04		0.04			
*W-W*	0.45		0.44			
W-R/R-W ratio	9.69		0.10			
Mean score change	0.032		−0.028			

*W-R* wrong to right response changes, *R-W* right to wrong response changes, *W-W* wrong to wrong response changes, *R-R* right to right response changes

^a^Percentage is calculated based on items with revisits

Table 4 breaks these same results down by examinee ability, as measured by total test scores. The high-ability examinees revisit more items than the low-ability examinees (17.9 ± 11.2 vs. 12.1 ± 8.3). It also appears that the high-ability examinees are slightly more likely to make a wrong to right change when they do change answers as reflected by the W-R/R-W ratio of 1.58 for the high-ability group and 1.28 for the low-ability group.

**Table 4:** Response change patterns and outcomes by examinee ability for block 4

	High Ability (*n* = 13,405)		Medium Ability (*n* = 11,917)		Low Ability (*n* = 2508)	
	Mean number of items	%	Mean number of items	%	Mean number of items	%
Items with revisits	17.9	40.7%	14.6	33.2%	12.1	27.5%
Items with revisits and no response change	16.5	92.2%^a^	13.2	90.4%^a^	10.7	88.4%
Items with revisits and response change	1.4	7.8%^a^	1.4	9.6%^a^	1.4	11.6%
*W-R*	0.63		0.56		0.51	
*R-W*	0.40		0.39		0.40	
*R-R*	0.04		0.03		0.03	
*W-W*	0.33		0.41		0.50	
W-R/R-W ratio	1.58		1.44		1.28	
Mean score change	0.005		0.004		0.003	

*W-R* wrong to right response changes, *R-W* right to wrong response changes, *W-W* wrong to wrong response changes, *R-R* right to right response changes

^a^Percentage is calculated based on items with revisits

Table 5 shows the results of the Multinomial Logistic Regression analysis. The likelihood ratio chi-square of 528.219 (*p* < 0.001) indicated that the model fits the data significantly better than a model with no predictors. Controlling for response duration, the odds of high ability examinees making a W-R (rather than R-W) response are 1.25 times higher than low ability examinees, while the odds of medium ability examinees making a W-R (rather than R-W) response are 1.11 times higher than low ability examinees.

**Table 5:** Results from the Multinomial Logistic Regression analysis for block 4

	Variable		Coefficient Estimate	Std. Error	OR	95% Confidence Interval	*P*-value
W-R vs. R-W log odds	Intercept		0.299	0.044			< 0.0001
	Ability:	High vs. Low	0.226	0.046	1.25	1.15–1.37	< 0.0001
		Medium vs. Low	0.103	0.046	1.11	1.01–1.21	0.026
R-R vs. R-W log odds	Intercept		−2.830	0.125			< 0.0001
	Ability:	High vs. Low	0.116	0.132	1.12	0.87–1.45	0.380
		Medium vs. Low	0.032	0.134	1.03	0.79–1.34	0.809
W-W vs. R-W log odds	Intercept		0.136	0.044			0.002
	Ability:	High vs. Low	−0.491	0.048	0.61	0.56–0.67	< 0.0001
		Medium vs. Low	−0.208	0.047	0.81	0.74–0.89	< 0.0001

*W-R* wrong to right response changes, *R-W* right to wrong response changes, *W-W* wrong to wrong response changes, *R-R* right to right response changes

We also used a linear mixed-effects model and examined how the amount of time spent revisiting items varied across different types of answer change, controlling for ability levels and item difficulty. The effects of answer change types, ability levels, item difficulty, and English proficiency on response duration were all significant (all *p* < 0.001). As listed in Table 6, the estimated marginal mean time spent reconsidering item responses was longest for the R-R changes (58.2 s), second longest for the W-W changes (50.4 s), and lowest for the R-W (46.4 s) and W-R changes (44.0 s). Post-hoc comparisons showed that response duration was significantly different for all answer change types as compared to R-W (all *p* < 0.001, after Bonferroni corrections).

**Table 6:** Item revisiting duration (in sec) across four types of response change patterns for block 4

	Item revisiting duration (Sec)		
	Marginal Mean^a^	Mean Difference from R-W (S.E.)	*P*-value
W-R	44.0	−2.4 (0.5)	< 0.0001
R-R	58.2	11.8 (1.4)	< 0.0001
W-W	50.4	4.0 (0.6)	< 0.0001
R-W	46.4	n/a	n/a

*W-R* wrong to right response changes, *R-W* right to wrong response changes, *W-W* wrong to wrong response changes, *R-R* right to right response changes

^a^Covariates appearing in the model are evaluated at the following values: STEP2 total test score = 232.51; Item difficulty = .7607

### Block eight analyses

As noted previously, in addition to block four we analyzed data from block eight to examine the stability of the results across the test day. All analyses were identical for block eight and the results were stable across the two blocks.

Table 7 summarizes the overall results. Again, most examinees (99.0%) revisited at least one item, and 69% of examinees changed at least one response (compared to 68% form block four). Among the examinees who changed responses 45.6% increased their scores and 26.8% lowered their scores (compared to 44.7 and 27.8% from block four).

**Table 7:** Response change patterns and outcomes for block 8

	Number of examinees	% (Based on all examinees)	% (Based on examinees with response change)
Examinees who revisited at least one item	27,521	99.0%	
Examinees who changed at least one response	19,178	68.9%	100.0%
*Examinees with score gain*	8742	31.4%	45.6%
*Examinees with score loss*	5142	18.5%	26.8%
*Examinees with unchanged score*	5294	19.0%	27.6%
	Mean number of items	% (Based on all items)	% (Based on items with revisits)
Items with revisits	15.8	35.9%	100.0%
Items with revisits and no response change	14.4	32.7%	91.1%
Items with revisits and response change	1.4	3.2%	8.9%
*W-R*	*0.60*		
*R-W*	*0.40*		
*R-R*	*0.03*		
*W-W*	*0.39*		
W-R/R-W ratio	0.60/0.40 = 1.50		
Overall mean score change	0.005		

*W-R* wrong to right response changes, *R-W* right to wrong response changes, *W-W* wrong to wrong response changes, *R-R* right to right response changes

On average each examinee re-visited 15.8 items (compared to 16 for block four). As with block four, on average each examinee changed responses to 1.4 of the re-visited items. Out of the 1.4 items with response changes, 0.60 responses were changed from wrong to right, 0.40 responses were changed from right to wrong, and 0.39 responses were changed from wrong to wrong. The W-R/R-W ratio was 1.50 (0.60/0.40). Again, these results are very similar to those from block four where the W-R/R-W ratio was 1.48 (0.59/0.40).

As with the overall results presented in Table 7 and in the previous paragraphs, the pattern of results for block eight closely parallel the results presented in Tables 3, 4, 5 and 6 (see Additional file 1: Tables S1–S4). These additional tables for block eight are available upon request.

## Discussion

Although a range of previous studies have examined the impact of changing answers and reported benefits from answer changes (e.g., [1, 2, 9, 14, 17, 24]), few studies examined the impact of changing answers in professionally developed, high-stakes, large-scale examinations [9, 24]. Of the studies that do focus on selection or credentialing tests, the Fischer et al. study is limited by the fact that responses of only 36 examinees were analyzed. This clearly provides a weak basis for generalizing beyond the studied sample. The present study expands previous research by analyzing responses from an annual cohort of over 27,000 examinees who completed the Step 2 CK examination. Broadly speaking, the results of this study are in line with the existing literature on answer changes. Most examinees appear to change answers, but on average they make relatively few changes. When examinees do change answers they tend to make more changes that increase their scores than those that lower their scores.

This paper substantially expands the information about how answer changes vary with examinee ability. Liu et al. [24] provides the only significant previous analysis of this relationship based on a large sample of examinees testing under high-stakes conditions. This paper also presents previously unavailable information about how the type of answer change relates to the amount of time examinees spend reviewing the changed items.

Results for answer changes conditioned on examinee ability suggest that more proficient examinees generally review more items, but they do not make more changes than less proficient examinees. When they do change answers, they have a greater likelihood of changing from wrong to right than less proficient examinees. Less proficient examinees review fewer items and are more likely to make wrong to wrong changes than more proficient examinees. This may be due to the fact that low ability examinees need more time to finish the examination and therefore may have limited time to revisit items. Regardless of ability level, changing answers generally results in benefit to the examinee overall.

While our results are in line with those from previous studies, the analyses presented in Tables 5 and 6, present new contributions to the literature in the analysis of how examinees allocate time in reviewing items for which they change answers. Examinees spend the most time (on average) considering items that they change from right to right and the second most on items changed from wrong to wrong, and shortest on items changed from right to wrong and wrong to right. That is, they allocate more time per item in considering changes that do not impact their scores. The fact that they spend more time considering the items they change from wrong to wrong is not surprising. These are clearly items for which the examinee does not know the correct answer and it stands to reason that they would spend time carefully reviewing the item text for hints. The right to right changes are harder to understand. One possible explanation is that the result is simply an artifact resulting from the fact that right to right changes require at least three actions (right to wrong and then wrong to right) and the other three types of changes can be accomplished with only two. These results may help examinees make decisions about how to allocate limited item review time when completing tests with time limits (such as most selection and credentialing examinations). Examinees should be aware of the fact that there is a point of diminishing returns related to time invested in reviewing a single item.

This study aimed to contrast the conventional wisdom (and popular advice) on answer changes and the empirical evidence. As with most previous studies, the results of this study support the view that answer changes generally benefit the examinee, regardless of the examinee’s ability level. This result would seem to argue against the conventional wisdom, but of course the examinee responses used in this study were, in some sense, influenced by that wisdom. In general practice, examinees are advised to exercise caution in changing answers. The relatively small number of items with answer changes may reflect that caution. It could be that the positive results from answer changes exist because examinees only change answers when they have considerable confidence that their changes will benefit their score. It is impossible to know how answer changes would impact scores in some counterfactual universe where the conventional wisdom did not exist. It may be that the current conservative approach to answer changing is an optimal strategy for the majority examinees, but it may also be the case that more answer changing would lead to greater improvements. Two conclusions do seem to be reasonable. First, excessive caution is not warranted because some levels of answer changing have consistently been show to improve scores (on average). Second, the size of the impact of answer changes is modest and at least in the present study it does not have an important impact on examinee scores or the resulting interpretations for most examinees.

## Additional files

**Additional file 1:** **Table S1.** Response change patterns and outcomes by score change for block 8. **Table S2.** Response change patterns and outcomes by examinee ability for block 8. **Table S3.** Results from the Multinomial Logistic Regression analysis for block 8. **Table S4.** Item revisiting duration (in sec) across four types of response change patterns for block 8.

## Data Availability

The data that support the findings of this study are available from National Board of Medical Examiners but restrictions apply to the availability of these data, which were used under license for the current study, and so are not publicly available. Data are however available from the authors upon reasonable request and with permission of National Board of Medical Examiners.
