# Supplementary material for: Investigation of answer changes on the USMLE® Step 2 Clinical Knowledge examination
Source: BMC Med Educ. 2019 Oct 23;19:389. doi: 10.1186/s12909-019-1816-3 (PMC6806526; doi:10.1186/s12909-019-1816-3)
Supplement: Supplementary file 1 — Additional file 1: Table S1. Response change patterns and outcomes by score change for block 8. Table S2. Response change patterns and outcomes by examinee ability for block 8. Table S3. Results from the Multinomial Logistic Regression analysis for block 8. Table S4. Item revisiting duration (in sec) across four types of response change patterns for block 8. [file 12909_2019_1816_MOESM1_ESM.docx]

Supplemental Table 1. Response change patterns and outcomes by score change for block 8.

|  | **Score Gain (n=8,172)** | | **Score Loss (n=5,142)** | | **No Score Change (n=13,894)** | |
| --- | --- | --- | --- | --- | --- | --- |
|  | **Mean number of items** | **%** | **Mean number of items** | **%** | **Mean number of items** | **%** |
| Items with revisits | 18.0 | 40.9% | 17.6 | 40.0% | 13.7 | 31.1% |
| Items with revisits and no response change | 15.8 | 87.8%* | 15.6 | 88.6%* | 13.0 | 94.9%* |
| Items with revisits and response change | 2.2 | 12.2%* | 2.0 | 11.4%* | 0.7 | 5.1%* |
| *W-R* | 1.54 |  | 0.15 |  |  |  |
| *R-W* | 0.16 |  | 1.37 |  |  |  |
| *R-R* | 0.04 |  | 0.04 |  |  |  |
| *W-W* | 0.46 |  | 0.44 |  |  |  |
| W-R/R-W ratio | 9.63 |  | 0.11 |  |  |  |
| Mean score change | 0.032 |  | -0.028 |  |  |  |

*Note: Percentage is calculated based on items with revisits.

W-R = wrong to right response changes; R-W = right to wrong response changes; W-W = wrong to wrong response changes; R-R= right to right response changes

Supplemental Table 2. Response change patterns and outcomes by examinee ability for block 8.

|  | **High Ability (n=13,405)** | | **Medium Ability (n=11,917)** | | **Low Ability (n=2,508)** | |
| --- | --- | --- | --- | --- | --- | --- |
|  | **Mean number of items** | **%** | **Mean number of items** | **%** | **Mean number of items** | **%** |
| Items with revisits | 17.6 | 40.0% | 14.5 | 33.0% | 12.2 | 27.7% |
| Items with revisits and no response change | 16.2 | 92.0%* | 13.1 | 90.3%* | 10.7 | 87.7% |
| Items with revisits and response change | 1.4 | 8.0%* | 1.4 | 9.7%* | 1.5 | 12.3% |
| *W-R* | 0.63 |  | 0.58 |  | 0.56 |  |
| *R-W* | 0.40 |  | 0.39 |  | 0.40 |  |
| *R-R* | 0.04 |  | 0.03 |  | 0.03 |  |
| *W-W* | 0.34 |  | 0.42 |  | 0.54 |  |
| W-R/R-W ratio | 1.58 |  | 1.49 |  | 1.40 |  |
| Mean score change | 0.005 |  | 0.004 |  | 0.003 |  |

*Note: Percentage is calculated based on items with revisits.

W-R = wrong to right response changes; R-W = right to wrong response changes; W-W = wrong to wrong response changes; R-R= right to right response changes

Supplemental Table 3. Results from the Multinomial Logistic Regression analysis for block 8.

|  | **Variable** | | **Coefficient Estimate** | **Std. Error** | **OR** | **95% Confidence Interval** | **P-value** |
| --- | --- | --- | --- | --- | --- | --- | --- |
| **W-R vs. R-W log odds** | Intercept | | 0.383 | 0.043 |  |  | <0.0001 |
|  | Ability: | High vs. Low | 0.127 | 0.045 | 1.14 | 1.04 - 1.24 | 0.005 |
|  |  | Medium vs. Low | 0.052 | 0.046 | 1.05 | 0.96 - 1.15 | 0.253 |
| **R-R vs. R-W log odds** | Intercept | | -2.668 | 0.116 |  |  | <0.0001 |
|  | Ability: | High vs. Low | -0.088 | 0.124 | 0.92 | 0.72 - 1.17 | 0.475 |
|  |  | Medium vs. Low | -0.034 | 0.125 | 0.97 | 0.76 - 1.23 | 0.784 |
| **W-W vs. R-W log odds** | Intercept | | 0.210 | 0.043 |  |  | <0.0001 |
|  | Ability: | High vs. Low | -0.550 | 0.047 | 0.58 | 0.53 - 0.63 | <0.0001 |
|  |  | Medium vs. Low | -0.252 | 0.047 | 0.78 | 0.71 - 0.85 | <0.0001 |

W-R = wrong to right response changes; R-W = right to wrong response changes; W-W = wrong to wrong response changes; R-R= right to right response changes

Supplemental Table 4. Item revisiting duration (in sec) across four types of response change patterns for block 8.

|  | **Item revisiting duration (Sec)** | | |
| --- | --- | --- | --- |
|  | **Marginal Mean*** | **Mean Difference**  **from R-W (S.E.)** | **P-value** |
| W-R | 42.6 | -2.9 (0.5) | <0.0001 |
| R-R | 57.3 | 12.7 (1.4) | <0.0001 |
| W-W | 48.4 | 3.8 (0.6) | <0.0001 |
| R-W | 44.6 | n/a | n/a |

* Covariates appearing in the model are evaluated at the following values: STEP2 total test score = 232.18; Item difficulty = .7573

W-R = wrong to right response changes; R-W = right to wrong response changes; W-W = wrong to wrong response changes; R-R= right to right response changes
